# Advantages of Blunt Radiofrequency Needles in CT‐Guided Dorsal Root Ganglion Pulsed Radiofrequency for the Treatment of Zoster‐Associated Pain

**DOI:** 10.1155/anrp/8776779

**Published:** 2025-11-09

**Authors:** Guanghui Zhang, Jing Yang, Xin Yao, Kai Luo, Zhiji Chen, Shuxiu Feng, Chunfang Wang, Xiaolan Gao, Zelin Weng

**Affiliations:** ^1^ Department of Pain Medicine, Second Affiliated Hospital of Xiamen Medical College, Xiamen, Fujian, China; ^2^ Department of Public Health, Second Affiliated Hospital of Xiamen Medical College, Xiamen, Fujian, China

**Keywords:** blunt radiofrequency needles, CT, dorsal root ganglion, pulsed radiofrequency, short-form McGill Pain Questionnaire, zoster-associated pain

## Abstract

**Objective:**

Compare the clinical efficacy of CT‐guided blunt versus sharp needles for dorsal root ganglion‐pulsed radiofrequency (DRG‐PRF) in treating zoster‐associated pain (ZAP).

**Method:**

This retrospective study analyzed 70 ZAP patients receiving CT‐guided DRG‐PRF between January 2021 and December 2024. Participants were divided into the blunt needle group (BNG, *n* = 30) and the sharp needle group (SNG, *n* = 40). Evaluated endpoints encompassed SF‐MPQ scores, postoperative analgesia use, intraprocedural trajectory pain (NRS), operative time, number of CT scan slices, and incidence of complications.

**Result:**

The BN group showed lower puncture NRS scores, shorter operative time, and fewer CT scan layers than the SN group (*p* < 0.05). SF‐MPQ scores (PRI, VAS, and PPI) decreased in both groups postoperatively (*p* < 0.05), with lower PRI and PPI in the BN group at Day 1, Week 1, and Months 1 and 3 (*p* < 0.05). Pregabalin use was lower in the BN group at Months 3 and 6, and tramadol use was lower at Week 1 and Month 1 (*p* < 0.05).

**Conclusion:**

CT‐guided DRG‐PRF demonstrates good analgesic efficacy in the treatment of ZAP. Compared with sharp needles, blunt needles show superior performance in terms of intraoperative puncture pain, procedure duration, radiation exposure, and postoperative medication use. As an optimized puncture instrument for DRG‐PRF, the blunt needle holds promise for broader clinical application and warrants further promotion.

**Trial Registration:**

Chinese Clinical Trial Registry: ChiCTR2500102560

## 1. Introduction

Zoster‐associated​ pain (ZAP) is a neuropathic pain syndrome caused by reactivation of varicella–zoster virus (VZV), resulting in damage to the dorsal root ganglion and sensory pathways [1]. It includes acute pain, subacute pain, and postherpetic neuralgia (PHN). It presents with diverse pain manifestations, tends to persist for a prolonged period, and often spans the entire natural course of the disease. Due to its intensity and refractory nature, the pain significantly disrupts patients’ daily activities and sleep, and may lead to the development of negative emotional responses such as anxiety and depression. As a result, ZAP markedly reduces patients’ quality of life and imposes long‐term adverse effects on their mental health [2].

For patients with ZAP, conventional medications such as gabapentin, pregabalin, and tramadol may provide some pain relief. However, due to their limited efficacy and potential side effects—including nausea, vomiting, nephrotoxicity, and the risk of dependence—nonpharmacological therapies are often required as part of a combined treatment approach [3]. In recent 10 years, image‐guided PRF targeting the DRG has emerged as a key interventional strategy in the management of ZAP [4, 5]. Nevertheless, during CT‐guided DRG‐pulsed radiofrequency (DRG‐PRF) procedures, the puncture pathway and choice of needle play a critical role in ensuring procedural safety. Sharp needles are commonly used to access the DRG target due to their ease of penetration; however, their pointed tip may cause direct intraoperative pain and potential nerve irritation when approaching nerve roots or ganglia, thereby increasing the risk of postoperative numbness and other complications [6]. The precise handling required for sharp needles also poses challenges for less experienced physicians. In contrast, blunt needles may reduce the risk of adverse events by minimizing direct puncture or compression of nearby structures such as nerves, blood vessels, and pleura during tissue traversal.

However, high‐quality evidence systematically comparing the multidimensional clinical value of CT‐guided blunt round needles versus sharp beveled needles for DRG‐PRF treatment of ZAP is currently lacking. Therefore, this study aims to compare the clinical effects of two different types of puncture needles in the interventional treatment of ZAP. Key outcome measures include intraoperative puncture pain, procedure duration, radiation exposure, postoperative pain assessed by the Short‐Form McGill Pain Questionnaire (SF‐MPQ), postoperative analgesic medication usage, and the incidence of complications. By systematically analyzing these multidimensional indicators, the study seeks to provide a more scientific and standardized technical basis for the interventional management of ZAP and to explore a safer, more effective, and patient‐friendly clinical pathway. The findings are expected to offer valuable evidence for optimizing future treatment strategies.

## 2. Materials and Methods

### 2.1. Study Design

A retrospective analysis was conducted on 70 patients with ZAP who underwent CT‐guided DRG‐PRF treatment at the Department of Pain Management, Second Affiliated Hospital of Xiamen Medical College, between January 2021 and December 2024. Based on the type of radiofrequency needle used, patients were divided into the sharp needle group (SNG, *n* = 30) and the blunt needle group (BNG, *n* = 40).

### 2.2. Inclusion and Exclusion Criteria

Inclusion criteria include the following: (1) age ≥ 18 years, no restriction on sex; (2) clinically diagnosed with ZAP [2], with a pain score (Numerical Rating Scale [NRS]) ≥ 4; (3) pain segment clearly localized and clinically evaluated as requiring CT‐guided DRG‐PRF treatment. In good general health, able to tolerate the procedure, and complete follow‐up. Exclusion criteria include the following: (1) presence of severe cardiovascular or cerebrovascular diseases, coagulation disorders, or infectious diseases; (2) local anatomical abnormalities or imaging indicating that critical structures may obstruct the puncture pathway; (3) pregnant or lactating women; (4) presence of severe psychiatric disorders or cognitive impairment preventing accurate pain assessment or follow‐up.

### 2.3. Materials

Sharp needle: A 150‐mm long, 10‐mm exposed tip, beveled straight needle manufactured by inomed Medizintechnik GmbH (registration number: National Medical Device Registration No. 20173157075), located at Im Hausgrün 29, 79,312 Emmendingen, Germany, was used.

**Figure 1:**
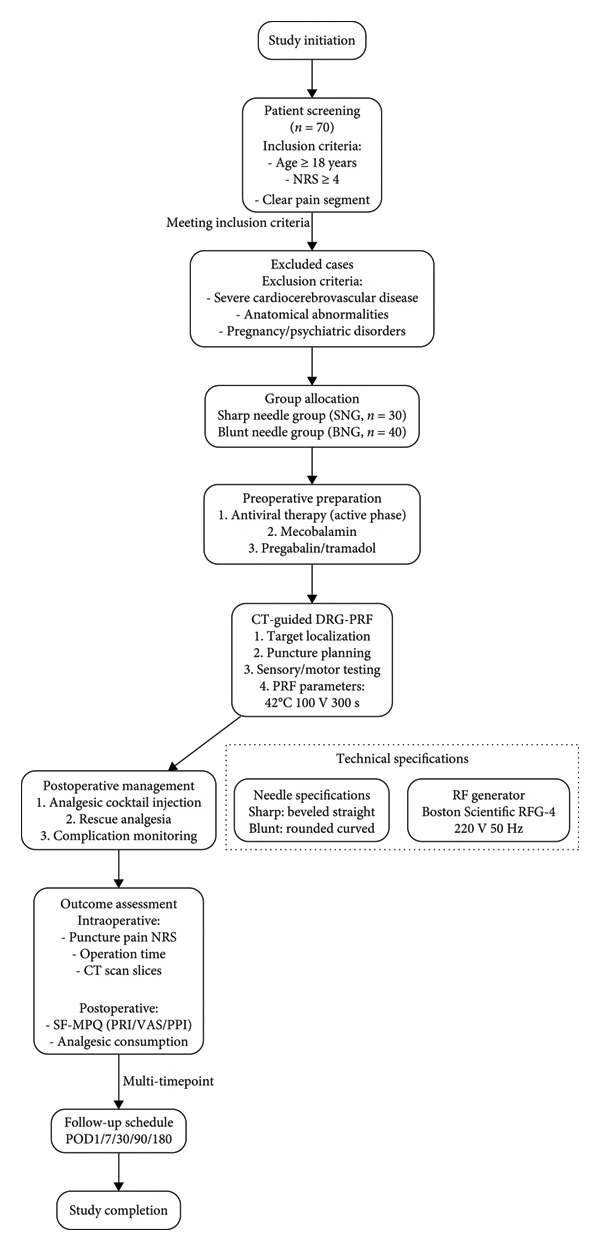
Flowchart of patient enrollment, grouping, and follow‐up procedures in the study.

Blunt needle: A 150‐mm long, 10‐mm exposed tip, blunt rounded curved needle produced by Beijing Beiqi Co., Ltd. (registration number: National Medical Device Registration No. 20223011283), located at Building 5, Four Seasons Sunshine Science Park, No. 375 Jushan Village, Haidian District, Beijing, China, was used (As shown in Figure 1).

Radiofrequency ablation device: The radiofrequency ablation device used in this study was manufactured by Boston Scientific Neuromodulation Corporation (Model RFG‐4‐220V, National Medical Device Registration No. 20153012776), located at 25,155 Rye Canyon Loop, Valencia, CA 91355, USA.

### 2.4. Methods

#### 2.4.1. Preoperative Management

Conventional pharmacological treatment: For patients in the active viral phase, oral treatment with famciclovir and mecobalamin tablets was administered. For patients in the subacute phase and beyond, mecobalamin tablets were given, with pregabalin and sustained‐release tramadol tablets administered orally as needed.

#### 2.4.2. Intraoperative Procedure

The patient was placed in the prone position, with standard monitoring of electrocardiogram, blood pressure, and oxygen saturation, and intravenous access was established. Based on the location of pain, the corresponding DRG segment for treatment was identified, and a positioning frame was placed in the target region. CT scanning was performed to confirm the target segment and puncture point.

After skin disinfection and sterile draping, local infiltration anesthesia was administered at the puncture site. A blunt needle or sharp needle was advanced along the preplanned path to the area adjacent to the lower margin of the pedicle near the DRG. After confirming that there was no cerebrospinal fluid or blood upon aspiration, the needle was connected to the radiofrequency generator for sensory and motor testing. The optimal needle tip position was defined as one that elicited sensory stimulation signs at 50 Hz and 0.3–0.6 V, but no motor response at 2 Hz and 0.6 V.

PRF treatment was then performed using the following parameters: temperature 42°C, voltage 100 V, frequency 2 Hz, pulse width 20 ms, and duration of 300 s. After PRF, 3 mL of anti‐inflammatory and analgesic solution (prepared with betamethasone injection 4 mg, mecobalamin injection 0.5 mg, 1% lidocaine 1 mL, and normal saline to a total of 8 mL) was injected at each segment.

Upon completion, the RF needle was withdrawn, and the following were recorded: puncture path pain score (NRS), operation duration, and the number of CT scan slices.

#### 2.4.3. Postoperative Follow‐Up and Treatment

For patients with unsatisfactory postoperative pain control, pregabalin and sustained‐release tramadol were administered to manage pain. The SF‐MPQ scores were recorded at postoperative Day 1 (POD1), Day 7 (POD7), Day 30 (POD30), Day 90 (POD90), and Day 180(POD180). Analgesic consumption (pregabalin and tramadol dosages) was documented at POD7, POD30, POD90, and POD180.

### 2.5. Observation Indicators

#### 2.5.1. Intraoperative Indicators

Puncture Path Pain Score: The pain experienced during needle puncture was assessed using the NRS.

Operation Duration (min): The total duration of the procedure was recorded.

Number of CT Scan Slices: The total number of CT slices was recorded from the initial localization scan to the final scan during the procedure.

#### 2.5.2. Postoperative Indicators

Pain Evaluation Using the SF‐MPQ: Pain levels in ZAP patients were assessed using the SF‐MPQ at predefined intervals: pretreatment baseline (d0), and posttreatment follow‐ups at Day 1 (POD1), Day 7 (POD7), Day 30 (POD30), Day 90 (POD90), and Day 180 (POD180). The SF‐MPQ evaluates three domains: Pain Rating Index (PRI; 0–45), Visual Analog Scale (VAS; 0–10), and Present Pain Intensity (PPI; 0–5), with higher scores indicating greater pain severity.

Postoperative Analgesic Use: Neuropathic pain medication consumption (including pregabalin and tramadol) was recorded at posttreatment Day 7 (POD7), Day 30 (POD 30), Day 90 (POD90), and Day 180 (POD 180). For nonstandard medications, doses were converted to pregabalin or tramadol equivalents.

#### 2.5.3. Safety Indicators

**Figure 2:**
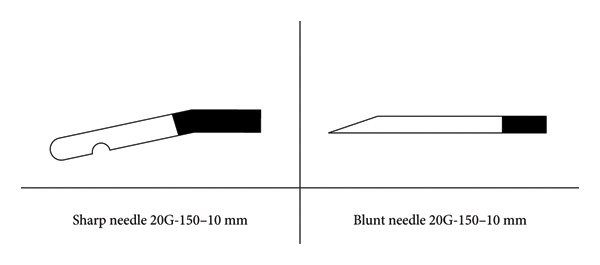
Technical specifications of the two types of radiofrequency needles used in the study.

Complication Rate: The incidence of complications such as intraoperative bleeding, infection, postoperative sensory abnormalities, and motor dysfunction was recorded (As shown in Figure 2).

### 2.6. Statistical Analysis

Statistical analysis was performed using SPSS 27.0. Normally distributed data were expressed as mean ± standard deviation (*x̄* ± *s*) and compared between groups using the independent samples *t*‐test, while within‐group comparisons were conducted using repeated measures analysis of variance. Non‐normally distributed data were presented as median (Q1, Q3) and compared using the Mann–Whitney *U* test. Categorical data were expressed as frequency (*n*) and analyzed with the *χ* ^2^ test or Fisher’s exact test. A *p* value < 0.05 was considered statistically significant.

## 3. Results

### 3.1. Baseline Characteristics

Among the 70 patients, 40 were treated with sharp needles (SNG) and 30 with blunt needles (BNG). The only difference between the two groups was the type of radiofrequency needle used. There were no statistically significant differences in baseline characteristics between the two groups, including sex, age, lesion location, disease duration, BMI, lesion side, preoperative VAS, PRI, and PPI scores, indicating comparability (*p* > 0.05, Table 1).

**Table 1:** Baseline characteristics.

Variables	SN group (*n* = 40)	BN group (*n* = 30)	*t/Z* value	*p* value
Gender (male/female)	21/19	15/15	0.043	0.836
Age (year)	63.93 ± 9.67	63.07 ± 10.13	0.360	0.720
Affected side (cervical/thoracic/lumbar)	3/33/4	6/22/2	2.489	0.288
Disease duration (week)	2.0 (1.0.5.0)	2.5 (1.0.5.0)	−0.108	0.914
BMI (kg/m^2^)	23.01 ± 3.29	22.55 ± 3.80	0.547	0.586
Affected side (left/right)	23/17	12/18	2.10	0.147
VAS (before treatment)	5.89 ± 1.02	5.88 ± 1.03	0.007	0.995
PRI (before treatment)	28.43 ± 5.40	28.83 ± 4.31	−0.341	0.734
PPI (before treatment)	3.42 ± 0.58	3.59 ± 0.48	−1.321	0.191

*Note:* Data are presented as mean ± SD or frequency.

Abbreviations: BMI, body mass index; PPI, present pain intensity; PRI, Pain Rating Index; VAS, Visual Analog Scale.

**Figure 3:**
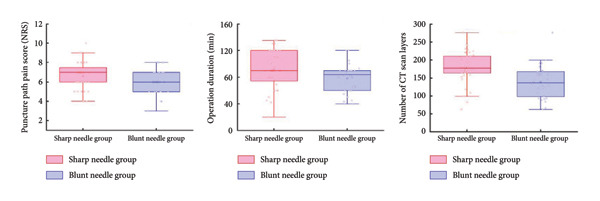
Intraoperative observations comparing CT scan layers, procedure time, and puncture‐related NRS scores between the blunt and sharp needle groups. Significant intergroup differences were observed in all three indicators.

Intraoperative evaluation items are described as follows (as shown in Table 2 and Figure 3).

1. The intraoperative NRS score for puncture pain was significantly lower in the SN group than that in the BN group (*p* = 0.049).
2. The operation time was significantly longer in the SN group than in the BN group (*p* = 0.031).
3. The number of CT scan slices was significantly higher in the SN group than in the BN group (median: 178 vs. 136.5, *p* < 0.001).

**Table 2:** Intraoperative evaluation items (mean ± SD).

	SN group (*n* = 40)	BN group (*n* = 30)	*t* value	*p* value
NRS score	6.63 ± 1.35	6.00 ± 1.20	2.004	**0.049** ^**∗**^
Operation time (min)	91.78 ± 28.38	78.00 ± 22.06	2.204	**0.031** ^**∗**^
Number of CT scan slices (median)	178.73 ± 42.99 (178)	139.23 ± 45.91 (136.5)	3.694	**< 0.001** ^**∗**^

*Note:* Data are presented as mean ± SD. Bold values denote statistical significance.

^∗^Significant as *p* value < 0.05.

**Figure 4:**
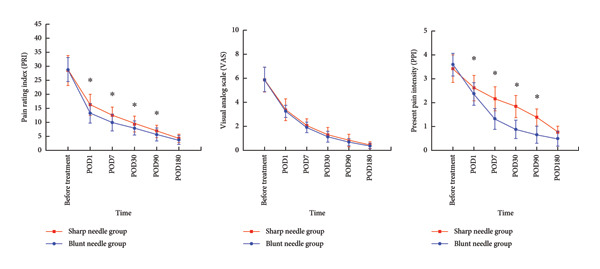
Short‐Form McGill Pain Questionnaire (SF‐MPQ) scores at multiple time points before and after pulsed radiofrequency.

SF‐MPQ scores are described as follows (as shown in Table 3 and Figure 4).

1. After treatment, analysis of PRI scores showed statistically significant changes over time within both groups (SN group: *F*_time_ = 253.760, *p* < 0.001; BN group: *F*_time_ = 310.873, *p* < 0.001). Intergroup comparisons indicated that patients in the SN group exhibited significantly higher PRI scores than those in the BN group at POD1 (*t* = 3.424, *p* = 0.001), POD7 (*t* = 3.774, *p* < 0.001), POD30 (*t* = 2.721, *p* = 0.008), and POD90 (*t* = 2.077, *p* = 0.042).
2. After treatment, analysis of VAS scores showed statistically significant changes over time within both groups (SN group: *F*_time_ = 322.391, *p* < 0.001; BN group: *F*_time_ = 318.726, *p* < 0.001). There were no significant differences in VAS scores between groups at any time point (*p* > 0.05).
3. After treatment, analysis of PPI scores showed statistically significant changes over time within both groups (SN group: *F*_time_ = 216.041, *p* < 0.001; BN group: *F*_time_ = 282.227, *p* < 0.001). Intergroup comparisons indicated that patients in the SN group exhibited significantly higher PPI scores than those in the BN group at POD1 (*t* = 2.105, *p* = 0.039), POD7 (*t* = 7.386, *p* < 0.001), POD30 (*t* = 9.179, *p* < 0.001), POD90 (*t* = 9.369, *p* < 0.001), and POD1800 (*t* = 4.212, *p* < 0.001).

**Table 3:** Comparison of SF‐MPQ scores before and after treatment between BN and SN groups (mean ± SD).

SF‐MPQ	Postoperative follow‐up time	SN group (*n* = 40)	BN group (*n* = 30)	*t* value	*p* value
PRI	Before treatment	28.43 ± 5.401	28.83 ± 4.31	−0.341	0.734
	POD1	16.25 ± 3.77	13.23 ± 3.48	3.424	**0.001**
	POD7	12.55 ± 2.93	9.87 ± 2.97	3.774	**< 0.001** ^**∗**^
	POD30	9.60 ± 2.55	7.93 ± 2.52	2.721	**0.008**
	POD90	6.80 ± 2.26	5.67 ± 2.26	2.077	**0.042**
	POD180	4.20 ± 1.49	3.73 ± 1.62	1.251	0.215
	*F* value	253.760	310.873		
	*p* value	**< 0.001** ^**∗**^	**< 0.001** ^**∗**^		

VAS	Before treatment	5.89 ± 1.02	5.89 ± 1.03	0.007	0.995
	POD1	3.38 ± 0.89	3.24 ± 0.50	0.835	0.407
	POD7	2.06 ± 0.56	1.91 ± 0.42	1.263	0.211
	POD30	1.30 ± 0.60	1.14 ± 0.44	1.286	0.203
	POD90	0.82 ± 0.48	0.68 ± 0.29	1.436	0.156
	*F* value	322.391	318.726		
	*p* value	**< 0.001** ^**∗**^	**< 0.001** ^**∗**^		
	POD180	0.46 ± 0.26	0.38 ± 0.23	1.325	0.190

PPI	Before treatment	3.42 ± 0.58	3.59 ± 0.48	−1.321	0.191
	POD1	2.62 ± 0.52	2.37 ± 0.47	2.105	**0.039**
	POD7	2.17 ± 0.50	1.33 ± 0.43	7.386	**< 0.001** ^**∗**^
	POD30	1.84 ± 0.46	0.89 ± 0.38	9.179	**< 0.001** ^**∗**^
	POD90	1.38 ± 0.36	0.66 ± 0.35	8.369	**< 0.001** ^**∗**^
	POD180	0.78 ± 0.26	0.50 ± 0.31	4.212	**< 0.001** ^**∗**^
	*F* value	216.041	282.227		
	*p* value	**< 0.001** ^**∗**^	**< 0.001** ^**∗**^		

*Note:* Data are presented as mean ± SD. Bold values denote statistical significance.

^∗^Significant as *p* value < 0.05.

**Figure 5:**
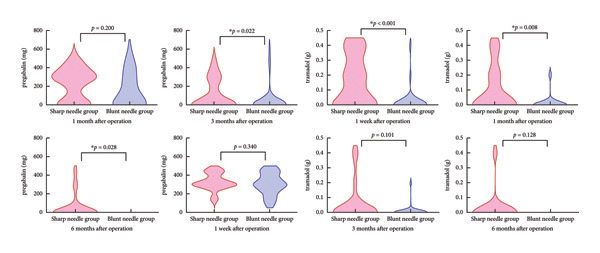
Postoperative consumption of pregabalin and tramadol on an as‐needed basis in both groups during follow‐up.

Analgesic drugs are described as follows (as shown in Table 4 and Figure 5).

1. Between‐group comparison of pregabalin usage showed that the SN group required a higher dose than the BN group at POM3 (*Z* = −2.283, *p* = 0.022) and POM6 (*Z* = −2.200, *p* = 0.028), while no significant difference was observed at POW1 and POM1.
2. Between‐group comparison of tramadol usage showed that the SN group required a higher dose than the BN group at POW1 (*Z* = −3.770, *p* < 0.001) and POM1 (*Z* = −2.670, *p* = 0.008), with no significant difference at POM3 and POM6.

**Table 4:** Posttreatment analgesic drugs usage between the two groups.

Analgesic drugs	Postoperative follow‐up time	SN group (*n* = 40)	BN group (*n* = 30)	*Z* value	*p* value
Pregabalin (mg)	POD7	300 (300, 431.25)	300 (225, 450)	−0.955	0.34
	POD30	300 (0.300)	112.5 (0.300)	−1.281	0.2
	POD90	0 (0.300)	0 (0.0)	−2.283	**0.022**
	POD180	0 (0.0)	0 (0.0)	−2.2	**0.028**

Tramadol (g)	POD7	0.1 (0.0.35)	0 (0.0)	−3.77	**< 0.001** ^**∗**^
	POD30	0 (0.0.2)	0 (0.0)	−2.67	**0.008**
	POD90	0 (0.0)	0 (0.0)	−1.642	0.101
	POD180	0 (0.0)	0 (0.0)	−1.522	0.128

*Note:* Data are presented as median (Q1, Q3). Bold values denote statistical significance.

^∗^Significant as *p* value < 0.05.

### 3.2. Complication

The incidence of complications was significantly higher in the SN group than that in the BN group (*p* = 0.034) (as shown in Table 5).

**Table 5:** Complication.

Complication	SN group (*n* = 40)	BN group (*n* = 30)
Intraoperative bleeding	3	0
Infection	0	0
Postoperative sensory abnormalities	3	0
Motor dysfunction	0	0
Total	6	0
*p* value	**0.034** ^**∗**^	

*Note:* The bold *p* value corresponds to the two‐tailed result of Fisher’s exact test.

^∗^Significant as *p* value < 0.05.

## 4. Discussion

Findings demonstrated that pain levels in both groups were significantly reduced from pretreatment through postoperative Day 1–Month 6, indicating that DRG‐PRF is an effective modality for managing ZAP. This finding is supported by multiple domestic and international studies [5, 7, 8]. A review by Wang et al. [9] also demonstrated that PRF can effectively alleviate ZAP by avoiding nerve injury through controlled temperature, with favorable efficacy and relatively few adverse events. In terms of intraoperative puncture‐related pain, complication rate, operation time, and CT radiation exposure, the BNG exhibited certain advantages.

Specifically, patients in the BNG reported significantly lower NRS scores during the puncture process from the subcutaneous layer to the DRG, compared with those in the SNG. This indicates that the blunt needle causes less pain stimulation during insertion, resulting in higher subjective comfort for the patient. This finding suggests that the blunt needle exerts less mechanical irritation and tissue trauma when traversing the skin, muscle layers, and perineural soft tissues. This advantage is likely attributable to the physical characteristics of the blunt needle tip. The blunt needle features a rounded, noncutting tip, which lacks the sharp penetrating force of beveled needles. As a result, it relies more on tissue separation rather than direct piercing during insertion, exhibiting a “blunt advancement” mechanism. When advancing through anatomical planes, the blunt needle conforms to the natural direction of tissue layers, minimizing direct injury to muscle fibers, small vessels, and sensory nerve endings, thereby reducing the perception of pain. Furthermore, the blunt tip is less likely to inadvertently puncture or damage the target nerve or adjacent structures, enhancing procedural safety and allowing the operator to make adjustments to the needle path more confidently under imaging guidance. Clinically, this gentle and anatomically conforming approach improves patient cooperation during the procedure, potentially reduces stress responses, and stabilizes intraoperative vital signs such as heart rate and blood pressure, thereby contributing to safer intraoperative management. In summary, the low‐stimulation and tissue‐sparing properties demonstrated by the blunt needle during puncture provide strong clinical support for its broader application in interventional pain management.

In terms of procedural efficiency, the SNG had significantly longer operative time and required more CT scan slices compared to the BNG, suggesting that the blunt needle offers advantages in both operational efficiency and radiation control. Given that CT‐guided interventional procedures require repeated scans for localization, the risks of radiation exposure to both patients and operators should not be overlooked. A study published by Yang et al. [10] in Radiology analyzed radiation doses in 9143 CT‐guided interventional procedures and reported substantial variations in dose across different types of interventions. For complex procedures, the median dose‐length product (DLP) could reach up to 2351 mGy cm, far exceeding that of routine diagnostic CT scans, indicating a significantly heavier radiation burden and highlighting the need for stricter dose management and optimization of procedural pathways.

The blunt needle used in this study, due to its high anatomical conformity and tissue‐dissecting capability, required fewer adjustments to the puncture trajectory during the procedure, which helped reduce repeated CT scans and thus lowered the patient’s radiation exposure. Additionally, the blunt tip of the needle minimizes the risk of accidentally puncturing blood vessels or critical structures, allowing the operator to perform the procedure more confidently and smoothly. This, in turn, reduces intraoperative hesitation and the frequency of repositioning, further shortening the operation time and radiation duration, thereby enhancing the overall safety and clinical applicability of interventional procedures.

This study further observed that, although the magnitude of VAS reduction was comparable between the two groups, PPI and PRI scores from postoperative Day 1–Month 3 were significantly lower in the BN group than those in the SN group. This discrepancy may be attributed to the distinct dimensions assessed by each scale: VAS primarily reflects pain intensity, whereas PRI and PPI also capture the qualitative characteristics of pain and patients’ subjective experiences. The use of blunt needles may reduce stimulation of tissues and nerves during the procedure, thereby preferentially improving pain quality and overall discomfort, which is reflected in PRI and PPI but not necessarily in VAS. In clinical practice, analgesic adjustments are often guided by VAS scores, aiming to maintain pain at a mild level that does not interfere with daily activities; this may partly explain the absence of significant differences in VAS outcomes between the groups. These findings further suggest that blunt needles not only minimize procedural trauma but also provide more pronounced short‐ to mid‐term analgesic benefits.

A meta‐analysis including 53 cohort studies evaluated the relationship between early treatment and the incidence of PHN. The results indicated that delayed treatment (≥ 3 days) was significantly associated with a higher incidence of PHN, underscoring the importance of early intervention in PHN prevention [11]. Timely pain control in the early stage may interrupt the vicious cycle of “pain–depression–sleep disturbance” and promote recovery through positive feedback [12]. Considering the potential link between early pain control in ZAP and subsequent chronic progression (e.g., PHN), the use of blunt needles in early interventions may hold extended clinical significance, warranting further investigation in studies with longer follow‐up durations.

The use of analgesic medications further supports the advantages of blunt needles. In this study, the BNG required less tramadol in the early postoperative period (1 week and 1 month) compared to the SNG, with no significant difference observed in the later postoperative period (3 and 6 months). As for pregabalin usage, there was no significant difference between the two groups in the early postoperative phase; however, in the later period, the BNG used significantly less, indicating a faster tapering of calcium channel modulators. These findings reinforce the superior efficacy of blunt needles.

**Figure 6:**
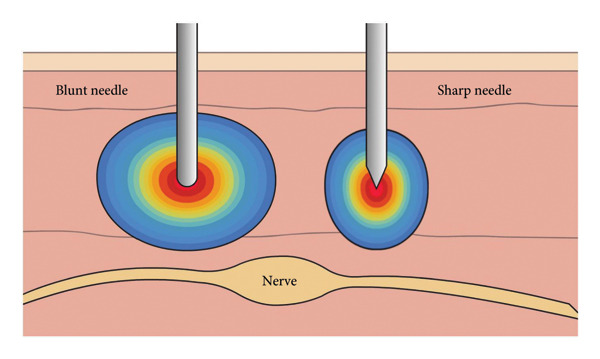
Schematic representation of electric field distribution around the needle tips during pulsed radiofrequency, showing differences between blunt and sharp RF needles.

This phenomenon can be attributed to the differences in the physical characteristics of the electric field generated by the two types of needle tips. The sharp needle, due to its pointed geometry and small electrode surface area, has a minimal tip curvature radius, which leads to a high concentration of electric charges at the needle tip, resulting in a “hot‐spot effect” in the electric field. This concentrated electric field yields a relatively narrow area of influence, making the efficacy of treatment more dependent on precise localization of the target. In contrast, the blunted radiofrequency needle, with its flat or rounded tip and larger electrode surface area, produces a more diffuse and evenly distributed electric field. The broader electric field coverage is better suited for modulating larger or less well‐defined target regions, such as widespread myofascial trigger points. In the context of pulsed radiofrequency treatment, which relies on nonthermal neuromodulation rather than tissue ablation, the broader distribution of the electric field offered by the blunted needle may facilitate more effective pain control (as shown in Figure 6).

In terms of safety, the BNG demonstrated a lower incidence of complications, suggesting that blunt needles exert less disturbance on local tissues and neural structures. The rounded, blunt tip of these needles is less likely to puncture nerves, blood vessels, or the pleura, making them suitable for high‐density operations near neural regions. This design helps reduce intraoperative dysesthesia, electric shock sensations, and other neural discomforts. In contrast, sharp bevel needles, while offering strong penetration and rigidity—allowing rapid traversal of ligaments and vessels—may cause greater tissue damage and irritation, increasing the risk of intraoperative discomfort and postoperative inflammatory responses.

The use of blunt needles in renal puncture procedures has demonstrated their effectiveness in reducing major renal artery injury and associated hemorrhage [13]. During DRG‐PRF procedures, the puncture path commonly traverses soft tissue near the transverse process or intervertebral foramen. The “gliding entry” feature of blunt needles minimizes mechanical trauma. This is particularly critical in cervical spine interventions, where vertebral artery injury is a highly dangerous complication. If such injury occurs, hemostasis is difficult, and the resulting hemorrhage or cerebral ischemia may be fatal [14].

In this study, two cases of postoperative numbness were observed in the SNG, possibly due to nerve contusion caused by the sharp needle tip contacting the nerve. Given the limited regenerative capacity of neural tissue, nerve injuries from sharp instruments tend to recover slowly. Blunt needles not only reduce this risk but also offer greater procedural confidence, especially for junior operators.

Current research on PRF for ZAP has primarily focused on optimizing treatment parameters, improving imaging guidance methods, or integrating techniques [15, 16]. A clinical study in 2024 found that high‐voltage pulsed radiofrequency provided superior analgesic effects without increasing the incidence of adverse events; however, its long‐term efficacy was not significant [8]. Wen et al. conducted a single‐center analysis of 63 patients with subacute PHN, comparing single versus repeated high‐voltage, long‐duration PRF treatments and reported that repeated treatments yielded higher overall effectiveness without serious adverse effects [17]. Some studies have also shown that patients with subacute herpes zoster–related neuralgia who receive ultrasound‐guided radiofrequency treatment experience better therapeutic outcomes [18]. Moreover, research indicates that dual guidance with ultrasound and C‐arm fluoroscopy, combined with stellate ganglion block, is an effective and cost‐efficient approach for managing acute herpes zoster–related neuralgia [19]. A review by Rui et al. highlighted the need for more high‐quality clinical studies to further optimize PRF parameters, explore novel combination therapies, and establish a more comprehensive complication prevention system [20].

However, systematic comparative studies on the selection of puncture needles—an element central to the efficacy of treatment—remain limited. As the key instruments that directly access the target lesion, puncture needles vary in design, material properties, and specifications, all of which can significantly influence clinical outcomes, procedural safety, and the efficiency of healthcare resource utilization. This study conducts a multidimensional comparative analysis of different puncture needles, providing robust empirical evidence through comprehensive clinical data and rigorous scientific evaluation. The findings offer valuable support for the rational selection of puncture tools and serve as an important supplement to the theoretical and practical framework of PRF treatment for ZAP.

This study also has certain limitations. First, although all procedures were performed by the same operator to minimize bias due to technical variability, differences in the operator’s familiarity with either the sharp needle or blunt needle may have influenced procedural performance and efficiency assessments, introducing potential operator bias. Second, the evaluation relied mainly on subjective or semisubjective measures, such as VAS scores, intraoperative records, and medication usage, lacking objective assessments of neural function. Future studies may incorporate imaging techniques and neurophysiological evaluations—such as nerve conduction studies or quantitative sensory testing (QST)—to further refine the assessment system.

In conclusion, CT‐guided DRG‐PRF appears to be an effective approach for the treatment of ZAP. In terms of needle selection, the blunt‐rounded needle demonstrated potential advantages over the sharp‐beveled needle with respect to procedural efficiency, safety, and postoperative patient comfort. Specifically, its use was associated with reduced puncture‐related pain, lower complication rates, shorter operation times, decreased radiation exposure, and improved postoperative pain relief. However, given the retrospective design and limited sample size of this study, these findings should be interpreted with caution, and further prospective studies with larger cohorts are warranted to validate these observations.

## Ethics Statement

This study was approved by the hospital’s ethics committee (Approval No.: 20251111). All procedures performed were in accordance with the ethical standards of the Institutional and National Research Committee and with the 1964 Helsinki Declaration and its later amendments.

## Consent

Written informed consent was obtained from all participants prior to their inclusion in the study. Written informed consent for publication was obtained from all participants whose data are included in this article.

## Disclosure

All authors reviewed and approved the final version of the manuscript.

## Conflicts of Interest

The authors declare no conflicts of interest.

## Author Contributions

Guanghui Zhang: conceptualization, methodology, investigation, and writing–original draft.

Jing Yang: resources, data collection, and writing–review and editing.

Xin Yao: methodology, project administration, and supervision.

Kai Luo: visualization, patient management, and investigation.

Zhiji Chen: clinical implementation and investigation.

Shuxiu Feng: formal analysis, data curation, and statistical validation.

Chunfang Wang: resources, data collection.

Xiaolan Gao: resources and follow‐up coordination.

Zelin Weng: supervision, writing–review and editing, and corresponding author.

## Funding

This research received no specific grant from any funding agency in the public, commercial, or not‐for‐profit sectors.

## Data Availability

The data that support the findings of this study are available from the corresponding author upon reasonable request.
